# Nasal symptoms increase the risk of snoring and snoring increases the risk of nasal symptoms. A longitudinal population study

**DOI:** 10.1007/s11325-020-02287-8

**Published:** 2021-01-19

**Authors:** Maria Värendh, Christer Janson, Caroline Bengtsson, Johan Hellgren, Mathias Holm, Vivi Schlünssen, Ane Johannessen, Karl Franklin, Torgeir Storaas, Rain Jõgi, Thorarinn Gislason, Eva Lindberg

**Affiliations:** 1 Department of Otorhinolaryngology, Head and Neck Surgery, Department of Clinical Sciences Lund, Skåne University Hospital, Lund University, SE-222 41 Lund, Sweden; 2 Department of Medical Sciences, Respiratory, Allergy and Sleep Research, Uppsala University, Uppsala, Sweden; 3 Department of Surgical Sciences, Otorhinolaryngology, Head and Neck Surgery, Uppsala University, Uppsala, Sweden; 4 Department of Otorhinolaryngology, Head and Neck Surgery, Sahlgrenska University Hospital, University of Gothenburg, Gothenburg, Sweden; 5 Occupational and Environmental Medicine, School of Public Health and Community Medicine, Institute of Medicine, Sahlgrenska Academy, University of Gothenburg, Gothenburg, Sweden; 6 Department of Public Health, Environment, Occupation and Health, Danish Ramazzini Centre, Aarhus University, Aarhus, Denmark; 7 The National Research Centre for the Working Environment, Copenhagen, Denmark; 8 Center for International Health, University of Bergen, Bergen, Norway; 9 Department of Surgical and Perioperative Sciences, Surgery, Umeå University, Umeå, Sweden; 10 Department of Occupational Medicine, Haukeland University Hospital, Bergen, Norway; 11 Lung Clinic, Tartu University Clinics, Tartu, Estonia; 12 Landspitali – The National University Hospital of Iceland, Reykjavík, Iceland; 13 Faculty of Medicine, University of Iceland, Reykjavík, Iceland

**Keywords:** Epidemiology, Nasal obstruction, Sleep, Snoring

## Abstract

**Purpose:**

Humans have a preference for nasal breathing during sleep. This 10-year prospective study aimed to determine if nasal symptoms can predict snoring and also if snoring can predict development of nasal symptoms. The hypothesis proposed is that nasal symptoms affect the risk of snoring 10 years later, whereas snoring does not increase the risk of developing nasal symptoms.

**Methods:**

In the cohort study, Respiratory Health in Northern Europe (RHINE), a random population from Denmark, Estonia, Iceland, Norway, and Sweden, born between 1945 and 1973, was investigated by postal questionnaires in 1999–2001 (RHINE II, baseline) and in 2010–2012 (RHINE III, follow-up). The study population consisted of the participants who had answered questions on nasal symptoms such as nasal obstruction, discharge, and sneezing, and also snoring both at baseline and at follow-up (*n* = 10,112).

**Results:**

Nasal symptoms were frequent, reported by 48% of the entire population at baseline, with snoring reported by 24%. Nasal symptoms at baseline increased the risk of snoring at follow-up (adj. OR 1.38; 95% CI 1.22–1.58) after adjusting for age, sex, BMI change between baseline and follow-up, and smoking status. Snoring at baseline was associated with an increased risk of developing nasal symptoms at follow-up (adj. OR 1.22; 95% CI 1.02–1.47).

**Conclusion:**

Nasal symptoms are independent risk factors for development of snoring 10 years later, and surprisingly, snoring is a risk factor for the development of nasal symptoms.

## Introduction

Healthy people breathe predominantly through the nose during sleep, and the resistance is lower than when breathing through the mouth [1]. Nasal breathing also has other advantages such as better ventilation compared to oral breathing [2]. Nasal symptoms such as obstruction can be a reason for promotion of an opening of the mouth. In patients with nasal obstruction, snoring is more frequent during nasal breathing than oral breathing [3], while apnoeas in patients with obstructive sleep apnoea (OSA) are more frequent during oral breathing than during nasal breathing [1]. In OSA, patients often breathe through the mouth during sleep [4]. An interesting pathophysiological question is whether or not there is a switch from nasal to oral breathing in the process where snoring and, later, OSA develop [5]. A few studies focus on aspects on the question. It has been shown in a cross-sectional study that nasal symptoms (nasal congestion, stuffy nose, sneezing, blocked nose, loss of smell, facial pain or sinus pressure, sore throat or hoarseness, and postnasal drip) increased the odds of having OSA [6]. It has also been described in a 5-year follow-up study (*n* = 4916) [7] that nocturnal nasal obstruction was a risk factor for snoring. It is not known if the direction of the association can be reversed; no studies have been located in the literature investigating the question.

In the current study, the aim was to investigate if nasal symptoms increase the risk of developing snoring and if snoring is a risk factor for the induction of nasal symptoms. The hypothesis proposed was that nasal symptoms have an impact on the risk of developing snoring, whereas snoring does not increase the risk of developing nasal symptoms.

## Material and methods

### Study design and study sample inclusive ethics

**Fig. 1:**
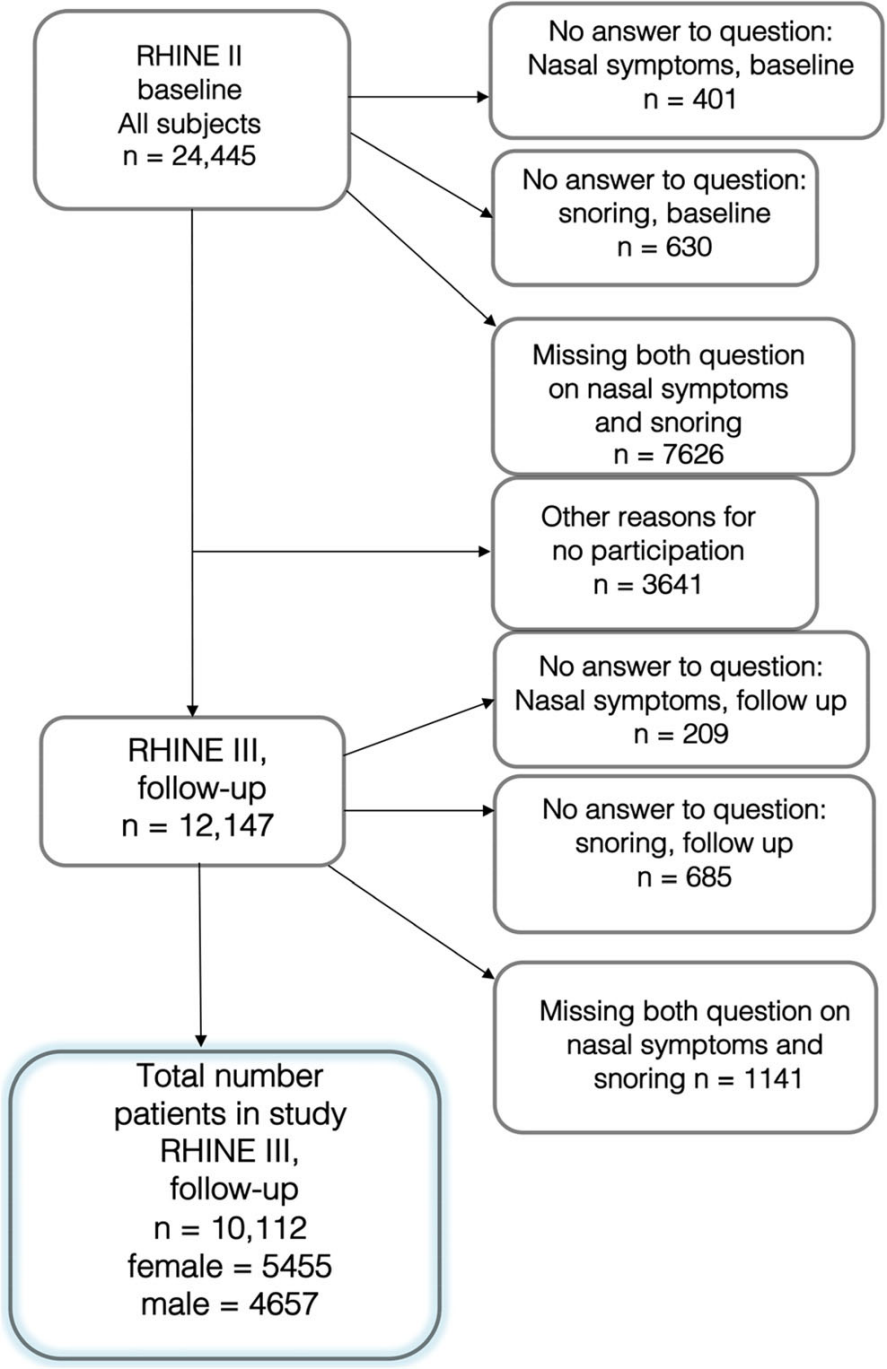
Flowchart overview of the study sample (*n* = 10,112)

This is an epidemiological study of a random population born between 1945 and 1973. The original cohort was part of the European Community Respiratory Health Survey (ECRHS) [8] stage I (www.ecrh.org). In a collaboration between the Northern European countries Denmark, Estonia, Iceland, Norway, and Sweden, a cohort called Respiratory Health in Northern Europe (RHINE) was developed (www.rhine.nu). The project is ongoing, and the cohort has been investigated at three different occasions so far, 1991–1993 (RHINE I), 1999–2001 (RHINE II), and 2010–2012 (RHINE III). Response rates at the different times have been described by Johannessen et al. [9]. The current study population consists of RHINE II (baseline) and RHINE III (follow-up) with the participants who answered questions on rhinitis and snoring at baseline and at follow-up. Patients without an answer to the questions on rhinitis and snoring at both baseline and follow-up were excluded from the analysis. The study population (*n* = 10,112) is outlined in Fig. 1.

All participants completed written informed consent at baseline and at follow-up. The local ethical committees at all sites of the study have approved the project.

### Definitions

The study participants were asked if they had loud and disturbing snoring, with the response alternatives being “Never”, “Less than once a week”, “Once or twice a week”, “Three to five nights/days a week”, or “Almost every night/day”. Having snoring was defined by reporting snoring ≥ 3/week. Having nasal symptoms was defined by two questions combined. The first question (1) was at baseline and at follow-up: answering “yes” to the question “Have you ever had nasal discomfort like nasal obstruction, nasal discharge and/or sneezing without having a cold?”. The second question (2) was on seasonal nasal symptoms and was investigated with the question “In which season are your symptoms the worst?” (Choose one alternative: “Spring”, Summer”, “Autumn”, “Winter”, “Always”, and “I do not know”). Having nasal symptoms were considered with the answer “Always” in question (2) as well as answering “yes” to question (1).

Self-reported weight and height were used to calculate body mass index (BMI) by the formula weight/(height in m)^2^, and the change in diff-BMI (Δ-BMI) was calculated by BMI at follow-up subtracted by BMI at baseline. The questions investigating smoking history were “Are you a smoker?” and “Are you an ex-smoker?”. In questions on comorbidity (i.e. cardiovascular and diabetes mellitus), the response alternatives were “Yes” or “No”.

Sleep apnoea was examined at follow-up with the questions “Have you ever been diagnosed with sleep apnoea by a doctor?” and “In what year were you diagnosed with sleep apnoea?”. Having OSA required being diagnosed at least 1 year prior to answering the questionnaire. If the study participants had been diagnosed prior to 1999 (baseline), they were considered having OSA at baseline.

### Data analysis (statistics)

Nominal data are presented as frequencies and percentages without decimals. Ordinal and quantitative data are presented by mean and standard deviation (mean ± SD). Non-parametric statistics were used to compare the differences between groups. The chi-square test was used in comparisons between nominal data in independent groups. The Mann–Whitney *U* test was used in two independent group comparisons of ordinal and quantitative data, and the Wilcoxon signed rank test was used when calculating paired group differences for 2 groups. Spearman’s rank correlation (rs) was used when measuring associations. In the binominal logistic regression analysis, the Enter method was used, which includes all predictors in the model. The statistical software used was SPSS 25.0 and STATA version 15.

## Results

### Study sample

The study sample (*n* = 10,112) is outlined in the flow chart in Fig. 1. Nasal symptoms were common; at baseline, 48% (*n* = 4903) of the study participants had nasal symptoms and 24% (*n* = 2435) reported snoring. General information about the participants is shown in Table 1. The mean age was 40.8 (± 7.2) years at baseline, 54% were women.

**Table 1:** General information about the participants. Characteristics at baseline in patients with either nasal symptoms or snoring at follow-up. At baseline, a high proportion of the study participants (*n* = 4903) reported nasal symptoms, and snoring was reported by 2435 participants

Baseline	All	Nasal symptoms (at follow-up)		*p*	Snoring (at follow-up)		*p*
	*n* = 10,112	No, *n* = 4903	Yes, *n* = 5209		No, *n* = 7608	Yes, *n* = 2504	
Age, mean ± SD	40.8 ± 7.2	41.0 ± 7.3	40.6 ± 7.2	*< 0.001*	40.4 ± 7.3	41.8 ± 6.9	*< 0.001*
Sex (female)	54%	52%	57%	*< 0.001*	59%	38%	*< 0.001*
BMI^1^ mean ± SD	24.6 ± 4.0	24.7 ± 4.1	24.6 ± 4.0	0.08	24.2 ± 3.9	26.1 ± 4.1	*< 0.001*
Smoking							
Never	25%	25%	26%	0.2	23%	31%	*< 0.001*
Previous	47%	48%	46%		49%	40%	
Current	25%	25%	26%		26%	27%	
Asthma	7%	2%	11%	*< 0.001*	6%	9%	*< 0.001*
Hay fever	24%	7%	41%	*< 0.001*	23%	25%	*0.04*
Nasal symptoms	48%	25%	72%	*< 0.001*	46%	53%	*< 0.001*
Snoring	18%	16%	19%	*< 0.001*	9%	47%	*< 0.001*
Sleep apnoea	1%	1%	1%	0.6	0.5%	2%	*< 0.001*
n-GER^2^	7%	6%	9%	*< 0.001*	6%	11%	*< 0.001*

^1^*BMI*, body mass index

^2^*n-GER*, nocturnal gastric oesophageal reflux

Significance in italics

Numbers given as mean ± standard deviation (SD) or as %

**Fig. 2:**
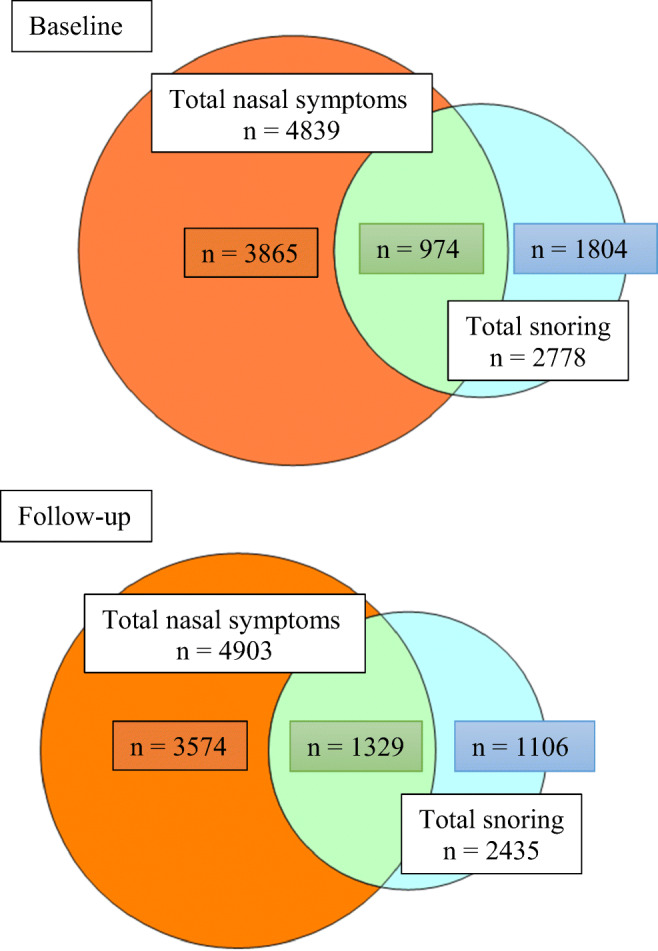
Proportions of patients with different symptoms at baseline and follow-up. Venn diagram showing the proportions of patients with nasal symptoms (orange), snoring (blue), and the combination of both nasal symptoms and snoring (green) at baseline. No snoring and no nasal symptoms were reported by *n* = 4353 at baseline. The next Venn diagram shows the same groups at follow-up

In Fig. 2, a Venn diagram shows the relationship between nasal symptoms and snoring at baseline and at follow-up. A large proportion of the patients with nasal symptoms also reported snoring.

**Fig. 3:**
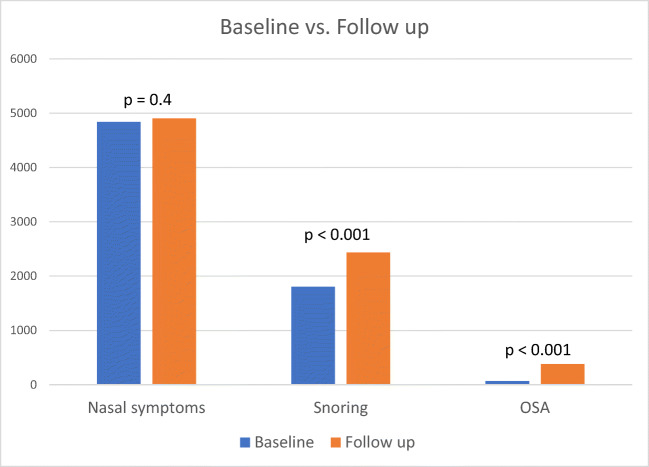
A comparison of nasal symptoms, snoring, and OSA at baseline vs. follow-up. OSA defined as diagnosed by a doctor and with a year of diagnosis. There was an increase in snoring (*p* < 0.001) and in OSA (*p* < 0.001)

After 10 years, there was an increase in the number of participants who reported snoring but no significant increase in nasal symptoms (Fig. 3).

Among the group that did not have nasal symptoms at baseline (*n* = 5273), new-onset nasal symptoms were reported by 1301 participants.

There was an increased risk of snoring at follow-up if nasal symptoms were present at baseline. The risk was increased by 38% compared to the group with no nasal symptoms at baseline. In a sensitivity analysis, with patients with OSA (*n* = 69) at baseline excluded, the unadjusted odds ratio (95% CI) for snoring was 1.37 (1.22–1.55).

For the patients who did not report snoring at baseline (*n* = 8308), snoring was reported by *n* = 1302 at follow-up. There was a 22% increased risk of new-onset nasal obstruction at follow-up if the participants reported snoring at baseline, see Table 3. When adjusted for the same variables as in Table 2, the odds ratio (95% CI) was 1.22 (1.04–1.49). In a sensitivity analysis, with patients with OSA at baseline excluded, the unadjusted odds ratio (95% CI) for nasal obstruction was 1.15 (0.97–1.37). When only including participants with nasal symptoms at baseline, snoring at baseline had an OR of 0.85 (95% CI 0.73–0.99) for nasal symptoms resolving at follow-up, and when controlling for the same variables as in Table 3, the adjusted OR was 0.86 (95% CI 0.23–1.01).

**Table 2:** Odds ratio of new-onset snoring if having nasal symptoms at baseline. Odds ratio of new-onset snoring at follow-up among participants with nasal symptoms at baseline was increased by 38% compared to the group with no nasal symptoms at baseline. Participants who had reported snoring at baseline were excluded

	Unadjusted odds ratio (95% CI) for snoring	Adjusted odds ratio (95% CI) for snoring
Nasal symptoms at baseline	*1.37* (1.22–1.54)	*1.38* (1.22–1.58)
Age	*1.02* (1.01–1.03)	*1.02* (1.02–1.03)
Age (38–49.9) (reference category)	1	1
Age (50–59.9) (reference category (38–49.9)	0.87 (0.73–1.04)	*1.52* (1.32–1.76)
Age (60–67) (reference category (38–49.9)	*1.25* (1.05–1.49)	*1.33* (1.10–1.61)
Sex (female)	0.54 (0.48–0.61)	0.49 (0.44–0.56)
Δ-BMI	*1.14* (1.11–1.17)	*1.16* (1.13–1.19)
Smoking previous	1.14 (0.98–1.32)	1.11 (0.95–1.29)
Smoking current	*1.44* (1.25–1.66)	*1.32* (1.14–1.54)

*CI*, confidence interval

Nasal symptoms defined as always having obstruction, nasal discharge, and/or sneezing without having a cold

Significance in italics. Multiple regression analysis was used

Odds ratio adjusted for sex, age, and previous and current smoking (difference in body mass index between baseline and follow-up)

**Table 3:** Odds ratio of new-onset nasal symptoms if snoring at baseline. Odds ratio of new-onset nasal symptoms at follow-up among participants with snoring at baseline was increased with 22% compared to the group with no nasal symptoms at baseline. All patients with nasal symptoms at baseline were excluded

	Unadjusted odds ratio (95% CI)	Adjusted odds ratio (95% CI)
Snoring at baseline	1.16 (0.98–1.38)	*1.22* (1.02–1.47)
Age	0.99 (0.98–1.00)	0.97 (0.97–0.99)
Age (38–49.9) (reference category)	1	1
Age (50–59.9) (reference category (38–49.9)	0.92 (0.81–1.06)	*1.27* (1.05–1.53)
Age (60–67) (reference category (38–49.9)	0.83 (0.69–0.97)	*1.13* (0.93–1.37)
Sex (female)	*1.17* (1.03–1.32)	*1.22* (1.07–1.40)
Δ-BMI	*1.04* (1.01–1.06)	*1.03* (1.01–1.06)
Smoking previous	1.17 (1.00–1.36)	1.13 (0.98–1.29)
Smoking current	*1.39* (1.19–1.61)	*1.39* (1.19–1.63)

*CI*, confidence interval

Nasal symptoms defined as always having obstruction, nasal discharge, and/or sneezing without having a cold

Significance in italics. Multiple regression analysis was used

Odds ratio adjusted for sex, age, Δ-BMI, and previous and current smoking (difference in body mass index between baseline and follow-up)

## Discussion

In this large longitudinal population study, the main findings are that nasal symptoms are risk factors for snoring and snoring on the other hand is also an independent risk factor for the development of nasal symptoms.

### Nasal symptoms are common and increase the risk of snoring

In the current study, almost half of the study subjects reported nasal symptoms at baseline, which is a similar number compared to 40% previously described by Hellgren et al. [10]. Nasal symptoms are common in the general population.

The current study shows that nasal symptoms increase the risk of snoring 10 years later.

The finding is in line with a previous epidemiological study by Young et al. [7] that found an increased risk of snoring 5 years after investigating nasal symptoms using a questionnaire at baseline. Very few recent studies investigate the relationship between nasal symptoms and snoring. One experimental study from 1991 [11] (*n* = 370) found that nasal airflow resistance before and after decongestant was a predictor of snoring index (snores per hour). One study by Virkkula [12] found (*n* = 37) a relationship between the combination of nasal obstruction and smoking and snoring but not for nasal obstruction alone. There are a small number of studies on treatment of nasal obstruction and effect on snoring. Koutsourelakis et al. [13] found an improvement in snoring frequency after treating nasal obstruction with nasal steroids. Surgical treatment of the nose has been tried to resolve snoring, but a review by Bury and Singh [14] concluded that while quality of life can be improved, the snoring is not cured. Positional therapy, weight loss, and mandibular advancement device have been concluded efficient in their review. These studies are focusing on other issues than the questions asked in the current study.

The relation between nasal symptoms and snoring is not thoroughly studied, but it is possible that having impaired nasal breathing will make the individual gradually sleep with the mouth open to compensate for the blocked nasal breathing. With mouth breathing, the person may start snoring more than when previously breathing through the nose while sleeping.

### Snoring increase the risk of nasal symptoms

On the other hand, it was surprising and opposite to our hypothesis that snoring increased the risk of nasal symptoms.

To our knowledge, no other study has shown that snoring can increase the risk of nasal symptoms. In a recent review on snoring [15], nasal symptoms were not mentioned among consequences from snoring.

It is interesting to speculate on possible explanations on the reasons for the worsening of the nasal symptoms in patients with snoring at baseline. In patients who have undergone laryngectomy and no longer use their nose at all, an objective decrease in nasal dimensions has been shown after 1 year [16]. It is possible that a subgroup of snorers is oral breathers despite normal nasal breathing, and years of low percentage of nasal breathing induce obstruction.

An inflammatory response has been shown in patients with OSA [17]. It could be speculated that an alternative explanation for the development of nasal obstruction in snorers is an inflammatory response in the airways as well as in the nose. On the other hand, a study by Liang Sun et al. [18] showed no relationship between increased biomarkers of inflammation and snoring, after controlling for BMI and waist circumference.

### Strengths and limitations

Major strengths of the current study are that the cohort is large and well described and the study participants included are living in five different countries.

The question at RHINE II defining nasal symptoms is a question asking if the patient has experienced nasal obstruction, nasal discharge, and/or sneezing. The patient can be experiencing only one of the symptoms and still answer “yes”, which means that a patient could have a nasal symptom but not a true rhinitis. According to Young et al. [7], only 39% of cases where severe nasal obstruction is maintained after 5 years are observed, and the validity is poor in determining the presence or absence of nasal obstruction. Even though in this study the number is 63%, there is a concern on whether it is valid to see a person who described nasal obstruction after 10 years of follow-up as a new-onset nasal symptom in a patient without nasal obstruction at baseline. This is an epidemiological study with limitations enclosed in the study method. There are no objective measures on the nasal symptoms or on the snoring or the occurrence of OSA. However, in a large cohort like this, tendencies can be evaluated to be investigated further in studies with other designs.

### Clinical implications

Snoring is common and is associated with the potential of reducing quality of sleep in patients and their bed partners. Snoring can also be the first step towards developing OSA.

An understanding of the pathophysiology of OSA is important to enable possible prevention of the development of the disease in the future.

It is important for general practitioners and ear, nose, and throat doctors as well as rhinologists to be aware of the risk of the development of nasal symptoms when patients are snorers. Doctors meeting patients with nasal problems should focus on quality of sleep as well as symptoms from the nose. With this knowledge, it is important to further investigate the relationship between nasal symptoms and snoring and thus prevent the development of future complications.

## Conclusions

Nasal symptoms are independent risk factors for the development of snoring, and snoring is a risk factor for the development of nasal symptoms.

## Data Availability

All datasets can be shared upon request.
